# Evidence-Based Practice and Its Associated Factors among Health Professionals Working at Public Hospitals in Southwest Ethiopia

**DOI:** 10.1155/2023/4083442

**Published:** 2023-12-13

**Authors:** Adamu Ambachew Shibabaw, Alex Ayenew Chereka, Agmasie Damtew Walle, Addisalem Workie Demsash, Shimels Derso Kebede, Ayenew Sisay Gebeyehu, Sefefe Birhanu Tizie, Daniel Niguse Mamo, Sisay Yitayh Kassie

**Affiliations:** ^1^Department of Health Informatics, College of Health Science, Mettu University, Mettu, Ethiopia; ^2^Department of Health Informatics, School of Public Health, College of Medicine and Health Science, Wollo University, Dessie, Ethiopia; ^3^Department of Health Informatics, School of Public Health, College of Medicine and Health Science, Debre Markos University, Debre Markos, Ethiopia; ^4^Department of Health Informatics, College of Medicine and Health Science, Arba Minch University, Arba Minch, Ethiopia

## Abstract

**Introduction:**

“Evidence-based practice” (EBP) is the process of incorporating clinical expertise and taking patient values and preferences into consideration when making clinical decisions. It is used to describe the provision of high-quality patient care.

**Objective:**

This study is aimed at assessing evidence-based practice and associated factors among health professionals working at public hospitals in Illu Aba Bora and Buno Bedele Zones, Oromia Region, Southwest Ethiopia, in 2022.

**Methods:**

An institution-based cross-sectional study design was conducted from May 8 to June 20 at public hospitals in Illu Aba Bora and Buno Bedele Zones, Oromia Region, Southwest Ethiopia. A total of 423 health professionals were included, using proportional allocation and simple random sampling. The data were collected using a self-administered questionnaire. Data was entered using EpiData version 4.6, and the collected data was cleared, arranged, coded, and then analyzed using Statistical Package for the Social Sciences version 26. Descriptive statistics and bivariable and multivariable analyses of logistic regression with AOR (95% CI) were performed at *p* < 0.05.

**Result:**

The study revealed that 36.2% of health professionals had good evidence-based practice. The factors found to be significantly associated with good EBP include having training in EBP (AOR = 5.43; 95% CI: 4.323, 9.532), good knowledge (AOR = 1.91; 95% CI: 1.065, 3.541), a favorable attitude (AOR = 1.91; 95% CI: 1.884, 2.342), and work experience greater than 5 years (AOR = 1.58; 95% CI: 1.482, 2.437).

**Conclusion:**

The evidence-based practice of health professionals was poor. Evidence-based practice should included in the curriculum, and also planned trainings need to be delivered to all health professionals, inorder to enhancing their knowledge as well as their attitudes by motivating them to increase evidence-based practice.

## 1. Introduction

“Evidence-based practice” (EBP) is the process of incorporating clinical expertise and taking patient values and preferences into consideration when making clinical decisions. It is used to describe the provision of high-quality patient care [1, 2]. Evidence is information that may be used by decision-makers in the healthcare industry and is derived from historical or scientific analyses of methods [3]. Its beginnings in medicine may be traced to the middle of the 19th century, when researchers began utilizing more advanced methodologies, statistical analysis, and results from natural science. Its importance grew as people's curiosity about the most effective medical treatments expanded [4, 5].

Evidence-based practice also equips medical professionals to enhance the standard of care by fusing the most recent research findings with innovative clinical problem-solving [6]. In addition to encouraging the development of theories and frameworks, it also establishes a national network of researchers to look into crucial advancements for all working healthcare professionals who support and employ the greatest information accessible when making clinical decisions [7–9]. The integration of the best research data, clinical skills, patient preferences, and desires will help health professionals bridge that gap and improve patient care, health, quality of service, quality of patient care, clinical efficacy, and nurses' productivity [10, 11].

The five processes that make up evidence-based practice include formulating a pertinent question, conducting an efficient literature search and study, critically evaluating the best available evidence, using the best evidence in clinical settings, evaluating care outcomes, and disseminating outcomes [12]. Evidence-based practice leads to many beneficial outcomes, including the provision of high-quality care, improved patient outcomes, and lower costs; however, it is not integrated into healthcare systems, and studies conducted globally discovered that the knowledge and skills of the healthcare workforce to use evidence-based practice were quite limited [13–15].

Evidence-based practice adoption was at 34.8% and 53%, respectively, among nurses and midwives working in various institutional settings in the Oromia Region, with nurses making up 61.5% of this population in the southern area. The prevalence of EBP was found to be much lower in sub-Saharan Africa, and Ethiopia has one of the lowest rates of EBP, with an estimated 46% of department heads using information in their decision-making [16–21]. Some of the factors that studies attempt to demonstrate are significantly associated with these low evidence-based practice problems at the individual level, including low levels of knowledge, a lack of familiarity with EBP, individual perceptions that support clinical decision-making, an inability to synthesize the available literature, and resistance to change [22].

The lack of infrastructure to access the literature, increased workload, lack of readily available research reports, and limited access to computers are organizational factors that were linked to the poor level of evidence-based practice implementation [23]. The evidence-to-practice gap still exists, according to the findings of multiple studies, and evidence-based practice guidelines from the West cannot be followed in Africa due to the low degree of evidence-based practice application among healthcare professionals [2, 24].

Due to a limitation of knowledge regarding the prevalence of evidence-based practice among health professionals and its contributing factors, a detailed investigation into various institutional structures and their working environments is necessary in Ethiopia [1, 18, 22]. To increase the motivation of health professional for better EBP, this study is essential for those who develop, plan, and implement EBP in health system. The results of this study will be used by program managers, stakeholders, and providers of healthcare services to improve EBP and raise the standard of treatment. The Federal Ministry of Health in Ethiopia needs to know the EBP of qualified health professionals. Additionally, there is little of an evidence-based culture throughout the health system in limited resources setting. This study evaluates evidence-based practice across all categories of health professional and identifies contributing factors. The findings of this study could help program managers, stakeholders, and health service providers improve EBP and quality healthcare services through intervention. It could also contribute to getting new research ideas for researchers. Therefore, this study was aimed at determining the proportion of evidence-based practice and identifying independent predictor variables of EBP among health professionals.

## 2. Method

### 2.1. Study Design and Period

The institutional-based cross-sectional study design was employed among health professionals in public hospitals in Illu Aba Bora and Buno Bedele Zones from November 8 to January 20, 2022.

### 2.2. Study Setting

The study was conducted at public hospitals in Illu Aba Bora and Buno Bedele Zones, Oromia Region, Southwest Ethiopia. The capital city of the Illu Aba Bora Zone is Mettu, whereas Bedele is the capital city of the Buno Bedele Zone. Mettu and Bedele cities are located 600 km and 471 km away from Addis Ababa, the capital city of Ethiopia, respectively. The two zones were demarcated as one administrative zone until recent times. The total population of those zones was 1,271,609. Among them, 636,986 and 634,623 were males and females, respectively. Farming is the predominant source of income in the community to support their lives. In terms of infrastructure development, there were 5 hospitals (1 referral hospital, 1 general hospital, and 3 primary hospitals) within the two zones. A total of 41 and 23 health centers were found in the Illu Aba Bora and Buno Bedele Zones, respectively.

### 2.3. Study Population and Eligibility Criteria

All healthcare professionals working in the public hospitals of Illu Aba Bora and Buno Bedele Zones and those who were found during the data collection period were the sources and study population, respectively. Healthcare professionals who were not permanently employed, those who were seriously ill, and those with work experience of less than four months were excluded.

### 2.4. Sample Size Determination

All health professionals permanently working in Illu Aba Bora and Buno Bedele Zones, Southwest Ethiopia, were eligible for this study. The sample size was calculated assuming the prevalence of healthcare providers' EBP practices to be 50% since the study was not focused specifically on EBP practices, similar to the current study setting. We also consider the following assumptions: a 95% level of confidence, a 5% margin of error, and a 5% nonresponse rate. Finally, a sample size of 423 was obtained. Five fully functional hospitals and 1,398 healthcare providers working in those hospitals were found in Illu Aba Bora and Buno Bedele Zones during the data collection period of the study. We proportionally allocated the total sample size of 423 to those five public hospitals found in the two zones.

### 2.5. Sampling Procedure

A simple random sampling method was used. Health professionals were randomly selected in those hospitals.

#### 2.5.1. Sampling Procedure and Sample Size

**Figure 1:**
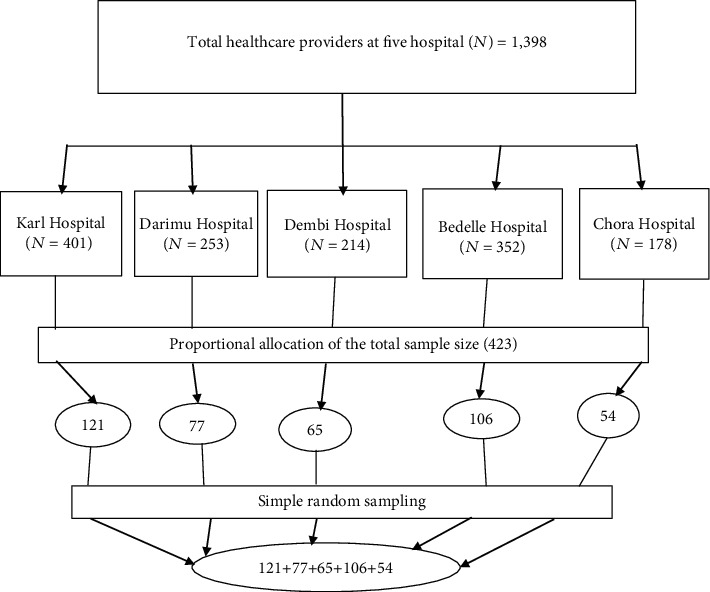
Sampling procedure of the study participant among health professionals working at public hospitals in Illu Aba Bora and Buno Bedele Zones, Southwest Ethiopia, 2022 (*n* = 409).

All five [5] hospitals located within the Illu Aba Bora and Buno Bedele Zones were approached and used for this study. All healthcare providers permanently working in those five hospitals were included in the study. The total sample size was proportionally allocated to each hospital. (1) The total sample size of 423 was allocated proportionally to all five hospitals found in our study setting. (2) Healthcare providers were randomly selected from those hospitals until we reached saturation based on the proportional allocation (Figure 1).

#### 2.5.2. Dependent Variable

The dependent variable for this study was evidence-based practice.

#### 2.5.3. Independent Variable

1. Sociodemographic characteristics: sex, age, educational status, working institution, work experience, and monthly income
2. Individual characteristics: participant knowledge of EBP and participant attitude towards EBP
3. Organizational characteristics: workload, Internet access, support from managers, working unit, and access of libraries are the organizational factors
4. Health professionals: in this study, health professionals were defined as those who have direct contact with patients, collect evidence-based patient information through direct assessment, and engage in evidence-based practice activities. Those included medical doctors, nurses, midwives, laboratory technicians, and others
5. Good knowledge: half and more than half of the questions answered by the respondents from the total knowledge-related questions were labeled as having “good knowledge” as well as “not good knowledge” [25]
6. Favorable attitude: respondents who scored above or equal to the mean for attitude assessment questions were also categorized as having a favorable attitude., otherways Unfavorable [25]
7. Good EBP: participants who scored above or equal to 60% for implementation assessment questions were considered to have “good EBP” [25]

### 2.6. Data Collection Tools and Procedures

The questionnaire has five parts. The first one is about sociodemographic characteristics; the second is a knowledge assessment question; the third is an attitude assessment question; the fourth is an EBP assessment question; and the last one is to address factors influencing the utilization of EBP. Questionnaires were adapted from different survey tools [25]. The questionnaire was prepared in English, and five health informatics were recruited for data collection. One health officer (HO) who has experience in research work supervised the data collection process. During data collection, the supervisors closely followed the day-to-day data collection process and ensured the completeness and consistency of the self-administered questionnaires each day before transferring them into computer software. Problems concerned with data collection were corrected early, and a nonoverlapping numerical code was given for each question to enter EpiData version 4.6.

### 2.7. Data Quality Assurance

One day of training was given for data collectors and supervisors on the objectives of the study, data collection procedures, data collection tools, respondents' approach, data confidentiality, and respondents' rights prior to the data collection date. The completeness of the questionnaire was checked every day by the supervisors. Data cleaning and cross-checking were done before the analysis. Before the actual data collection, the pretesting of the questionnaire was checked. The pretest was done with participants working in a similar environment outside of the study area. While performing the pretest among 5% (*n* = 21) of the sample, we calculated a Cronbach alpha and found its value to be 0.84. The actual data collection questionnaire was started after the necessary corrections.

### 2.8. Data Management

The collected data was entered manually into EpiData version 4.6 for cleaning, editing, organizing, and checking completeness. Then, it was stored in electronic databases and the Internet cloud to prevent data loss. Finally, the data was exported to SPSS version 26.

### 2.9. Statistical Analysis

After data collection, the response was checked and entered into a computer using EpiData version 4.6, and SPSS version 26 was used for data analysis. Descriptive statistics, including frequencies and percentages, were calculated for all variables, and those were presented in the form of tables, text, and graphs. Bivariable logistic regression analysis was carried out to see the association between the outcome and each explanatory variable, and then, variables with a *p* value of 0.2 were selected for multivariable logistic regression analysis. Variables having a *p* value < 0.05 in multivariable logistic regression analysis were used to declare statistical significance. Before running the logistic regression model, the assumptions of multicollinearity were checked and showed all variance inflation factor (VIF) values less than three, which demonstrated the absence of multicollinearity. Finally, the Hosmer and Lemeshow test was used to measure model fitness, with a *p* value of > 0.05 considered statistically significant, and it was 0.34.

## 3. Result

### 3.1. Sociodemographic Characteristics of Healthcare Professionals

The response rate was 96.7%; 409 out of 423 study participants responded. More than half (237, or 58.00%) of the respondents were male. The mean age was 30.05, with a standard deviation of 5.4 years. Almost half of the respondents (49.40%) were married. The majority of the professionals (52.1%) were nurses; 58.2% had a bachelor's degree; and 56.7% had less than 5 years of work experience (Table 1).

### 3.2. Healthcare Professional Attitude towards Evidence-Based Practice

On the Likert scale, 42.3% of the respondents had a favorable attitude towards evidence-based practice. Most of the respondents (42.5%) strongly agreed that implementing EBP improves care. Majority of the respondents (41.0%) agreed that critically appraising evidence is an important step during EBP; however, 94 (23.0%) of the respondents disagreed that clinical decision-making practice based on evidence is time-saving (Table 2).

### 3.3. Evidence-Based Practice (EBP) of Healthcare Professionals

**Figure 2:**
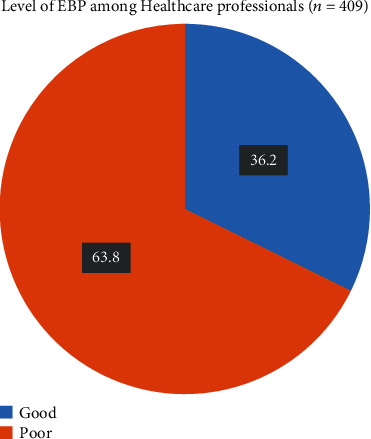
Level of EBP among health professionals working at public hospitals in Illu Aba Bora and Buno Bedele Zones, Southwest Ethiopia, 2022 (*n* = 409).

The evidence-based practice among health professionals working at public hospitals in Illu Aba Bora and Buno Bedele Zones was 36.2% with a 95% CI (30.8–38.9). Majorities of them—144 (35.6%)—used systematic review reports in clinical practice once to three times in the previous two months. Furthermore, 127 (31.4%) of the study participants evaluated their clinical practice based on scientific explanation, and 149 (36.8%) used textbooks, hospital protocols, WHO guidelines, or national guidelines to make decisions 4-6 times in the two months prior to this study. Moreover, 125 (30.9%) of participants changed their practice based on patient outcome data, and 141 (34.8%) of participants shared currently available evidence with multidisciplinary team members (Figure 2).

### 3.4. Organizational-Related Characteristics

Among the 409 study participants, 275 (67.2%) health professionals said that hospital facilities lack computer access to utilize EBP, and 285 (69.7%) professionals reported that a lack of Internet access made it difficult to use the current best evidence for clinical decision-making. As a result, 85.6, 82.4, 78.0, and 74.1% of participants reported that there was a lack of library access to practice EBP, a lack of training about evidence-based practices, managers who do not support the use of evidence-based practice, and a lack of authority in the workplace to change practices, respectively (Table 3).

### 3.5. Individual Characteristics Associated with EBP

On bivariate analysis, ten variables showed evidence of some association with the outcome at a *p* value of < 0.2, hence being included in the multivariate logistic regression analysis. From those variables, EBP training, attitude towards EBP, working experience, and knowledge level were statistically significant for EBP. Healthcare providers who received EBP training were about 5.43 times more likely to use EBP effectively than those who did not (AOR = 5.43; 95% CI: 4.323, 9.532). Healthcare providers who had good knowledge of EBP were 1.91 times more likely to use EBP effectively (AOR = 1.91; 95% CI: 1.065, 3.541) than those who had poor knowledge. Favorable attitude towards EBP was 1.91 times higher among healthcare providers with an AOR of 1.91 (95% CI: 1.884, 2.342) than among those with an unfavorable attitude. Working experience greater than 5 years of EBP was 1.18 times higher among healthcare providers with an AOR of 1.58 (95% CI: 1.482, 2.437) than among those with less than 5 years of working experience (Table 4).

## 4. Discussion

The evidence-based practice among health professionals working at public hospitals in Illu Aba Bora and Buno Bedele Zones was 36.2%, with a 95% CI of 30.8 and 38.9. It is consistent with studies conducted in the Oromia Region's public hospitals among nurses and midwives that revealed 34.8% of nurses and midwives practice evidence-based practices [25].

On the other hand, the result of this study is lower than a study conducted in regional hospitals in Taiwan, which found that 42% of participants had implemented EBP for clinical decision-making [26]. The difference might be due to sociodemographic variation, health system structure, and availability of different infrastructure that supports evidence-based practice, such as the Internet, computers, and smart phones. Furthermore, policymakers' commitment to implementing evidence-based practices varies from country to country [26].

This study was also lower than that of a study conducted in Black Lion Hospital 42.4% and in Northwest Ethiopia 53% of health professionals who used EBP in their clinical decision making. The difference might be due to the fact that previous studies were conducted in Black Lion Hospital, which has infrastructure that supports evidence-based practice, such as computers, Internet access, and training resources. Findings to apply evidence-based practice in public hospitals, there is no training conducted about evidence-based practices by government agencies, and there is a lack of updated guidelines and resources.

On the other hand, this finding was higher than a study conducted on barriers to nurses' participation in and utilization of clinical research in Ghana, which was 25.3%. The difference might be attributed to the fact that the previous study only included nurses, whereas this study included different healthcare providers, so there might be differences in knowledge about evidence-based practice.

The result of this study showed that male sex, EBP training, attitude about EBP, working experience, and knowledge had a significant effect on evidence-based practice. Male healthcare professionals were 1.85 times more likely to practice evidence-based decision-making than female participants. A study conducted at public hospitals in Jimma, Southwest Ethiopia, and provided support for this study. The possible reason for this association might be that females usually have multiple responsibilities at home, which make them busy and leaves them with a lack of time for searching for new evidence. It is also due to the fact that women are less involved in training and managerial positions in developing countries. Healthcare providers who received EBP training were about 5.43 times more likely to use EBP effectively than those who did not. Other studies' findings support this result: EBP training enables health professionals to implement evidence-based practice in their daily clinical services and is a significant predictor of EBP competence, knowledge, attitude, and utilization. Moreover, the study has shown that the knowledge of health professionals is significantly associated with EBP implementation. The odds of having good EBP were 1.91 times higher among healthcare providers with good EBP knowledge than among those with poor knowledge. Likewise, having good knowledge about EBP was positively associated with the utilization of evidence-based practice in the study at Gondar and the systematic reviews from low- and middle-income countries. Similarly, in our country, one study that was conducted at Black Lion Hospital reported that those health professionals who had knowledge were three times more likely to practice evidence-based medicine than those who did not have knowledge. The reason behind these similarities might be due to the link between knowledge and practice, as the chance of exercising something after knowing it is better in the majority of circumstances when applying evidence-based practice. Professionals who had a favorable attitude were 1.91 times more likely to practice evidence-based medicine than those who did not have a favorable attitude. This study is supported by the study done by midwives working in Amhara Region government hospitals. This was due to the attitude boost from the EBP. Professionals who have work experience greater than 5 years are 1.58 times more likely to practice evidence-based medicine than those with less than 5 years' experience. This study was supported by the study done in southern Ethiopia. This was due to work experience increasing professional attitude and skill for EBP.

## 5. Strength and Limitation

This study is the first attempt at all five hospitals. The study used a self-administered questionnaire, so most of the variables might have been exposed to social desirability bias. However, we recruited data collectors outside of the study hospitals (who were not members of the study hospitals), and we believed that we minimized the bias with our maximum effort.

## 6. Conclusion

In general, the evidence-based practice among health professionals working at public hospitals in Illu Aba Bora and Buno Bedele Zones was low. EBP should be included in the curriculum of medical and health science students. These study's findings offer valuable information about evidence-based practice and the barriers to evidence-based practice in public hospitals. In this study, the overall evidence-based practice and factor affecting EBP at public hospitals in the Illu Aba Bora and Buno Bedele Zones were examined.

## 7. Recommendations

All efforts shall be made to conduct training sessions for all health professionals. Therefore, this fosters the evidence-based practice knowledge of professionals. Institutions should emphasize enhancing health professionals' knowledge through training. Managers need to be dedicated and able to create good knowledge and attitude for the team members frequently to establish a well-established and profound evidence-based practice. We recommend that upcoming researchers study the evidence-based practice of health professionals using comparative studies to know and evaluate the quality of healthcare services between private and public hospitals.

## Figures and Tables

**Table 1:** Sociodemographic characteristics of healthcare professionals, 2022 (*n* = 409).

Variables	Frequency (*n*)	Percent (%)
Age		
20-29	165	40.34
30-39	183	44.74
≥40	61	14.91
Sex		
Male	237	58.00
Female	172	42.00
Marital status		
Single	172	42.05
Married	202	49.40
Divorced	35	8.60
Profession		
Physician	72	17.6
Nurse	213	52.1
Midwifery	61	15.00
Laboratory	35	8.6
Pharmacy	28	6.8
Educational level		
Diploma	70	17.11
Bachelor's degree	238	58.2
Master of science	34	8.3
General physician	35	8.56
Specialist	32	7.8
Working hours per day		
≤8	262	64.05
>8	147	35.9
Work experience		
≤5 years	232	56.7
>5 years	177	43.3
Monthly income		
<10,000 ETB	352	86.06
10,000-14,999	38	9.3
≥15,000	19	4.65

**Table 2:** Healthcare professionals' attitude towards evidence-based practice working at public hospitals in Illu Aba Bora and Buno Bedele Zones, Southwest Ethiopia, 2022 (*n* = 409).

Items	Strongly disagree	Disagree	Neutral	Agree	Strongly agree
Implementing EBP improves the care that I deliver to my patients	4 (0.9)	5 (1.2)	40 (9.8)	186 (45.5)	174 (42.5)
Evidence-based practice is relevant to my profession	3 (0.7)	3 (0.7)	34 (8.3)	201 (49.1)	168 (41.0)
Critically appraising evidence is an important step in the EBP process	4 (0.9)	10 (2.5)	55 (13.4)	220 (53.8)	120 (29.3)
Research articles from trusted sources are relevant to daily practice	5 (0.2)	18 (4.4)	75 (18.3)	181 (44.3)	130 (31.8)
Comments provided by colleagues that are based on established evidence could be accepted	11 (2.7)	19 (4.7)	92 (22.5)	171 (41.8)	116 (28.4)
Clinical decision-making practice based on evidence is time-saving	15 (3.7)	42 (10.4)	82 (20.0)	176 (43.0)	94 (23.0)

**Table 3:** Organizational-related factors for evidence-based practice among healthcare professionals working at public hospitals in Illu Aba Bora and Buno Bedele Zones, Southwest Ethiopia, 2022 (*n* = 409).

Variables	Yes *n* (%)	No *n* (%)
Lack of computer access to practice EBP	275 (67.2)	134 (32.8)
Lack of Internet access to practice EBP	285 (69.7)	124 (30.3)
Lack of access in new treatment guidelines to practice EBP	188 (46.0)	221 (54.0)
Lack of library access to practice EBP	350 (85.6)	59 (14.4)
Lack of training about evidence-based practice	337 (82.4)	72 (17.6)
Difficulty in understanding English to use the literature	122 (27.7)	287 (70.2)
Inability to implement recommendations of research studies into clinical practice	153 (37.4)	256 (62.6)
Difficulty in judging the quality of research papers and reports	192 (46.9)	217 (53.1)
Difficulty in determining the applicability of research findings	178 (43.5)	231 (56.5)
No sufficient time to find new guidelines/protocols online	236 (57.7)	173 (42.3)
Difficult to understand recent national treatment guidelines and protocols	143 (35.0)	266 (65.0)
The culture of your team is not receptive to changing practice	202 (49.4)	207 (50.6)
Lack of authority in the workplace to change practice	303 (74.1)	106 (25.9)
Managers do not support the use of evidence-based practice	319 (78.0)	90 (22)
No interdisciplinary discussion during patient management	248 (60.6)	161 (39.4)

**Table 4:** Bivariable and multivariable logistic regression analyses for EBP at public hospitals in Illu Aba Bora and Buno Bedele Zones, Southwest Ethiopia, 2022 (*n* = 409).

Variables	EBP		COR (95%)	AOR (95%)
	Good (%)	Poor (%)		
Age				
20-29	41 (30.8)	119 (43.2)	1	1
30-39	64 (48.5)	126 (45.8)	0.67 (0.512, 1.157)	1.65 (0.323, 1.315)
≥40	28 (20.8)	31 (11.2)	0.38 (0.103, 1.316)	2.51 (0.174, 1.497)
Gender				
Female	49 (36.3)	130 (47.4)	1	1
Male	86 (63.7)	144 (52.5)	0.63 (0.042, 2.623)	1.85 (1.021, 1.907)^∗^
Marital status				
Single	41 (30.6)	125 (45.1)	1	1
Married	18 (13.4)	129 (46.6)	2.35 (1.052, 2.370)	3.52 (0.819, 2.804)
Divorced	18 (13.4)	23 (8.3)	0.42 (0.286, 1.432)	1.12 (1.845, 5.337)
Profession				
Pharmacy	12 (8.8)	17 (6.1)	1	1
Physician	33 (24.3)	34 (12.1)	0.73 (0.568, 3.500)	0.11 (0.202, 2.523)
Nurse	59 (43.4)	160 (57.1)	1.91 (0.234, 2.218)	2.76 (0.278, 2.099)
Midwifery	22 (16.2)	42 (15.0)	1.35 (1.294, 1.889)	3.95 (0.305, 2.931)
Laboratory	10 (7.4)	27 (9.6)	1.91 (0.171, 2.481)	4.62 (0.169, 2.249)
Specialty				
Bachelor's degree	70 (52.3)	152 (55.3)	1	1
Master of science	14 (9.8)	28 (9.9)	0.92 (0.616, 4.660)	1.06 (1.322, 1.494)
General physician	22 (15.9)	19 (6.6)	0.43 (0.413, 16.113)	4.98 (1.333, 4.706)
Taking EBP training				
No	76 (58.0)	249 (91.6)	1	1
Yes	66 (42.0)	35 (8.4)	0.16 (0.065, 4.341)	5.43 (4.323, 5.532)^∗∗^
Knowledge about EBP				
Poor	75 (53.6)	80 (29.4)	1	1
Good	65 (46.4)	191 (70.6)	2.78 (1.742, 3.367)	1.91 (1.065, 3.541)^∗∗^
Attitude about EBP				
Unfavorable	81 (57.6)	124 (45.6)	1	1
Favorable	60 (42.4)	148 (54.4)	1.61 (1.156, 2.553)	1.91 (1.884, 2.342)^∗^
Working hrs				
≤8	61 (46.5)	71 (28.0)	1	1
>8	70 (53.5)	181 (72.0)	2.22 (0.285, 2.982)	0.45 (0.285, 0.982)
Working experience				
≤5 years	63 (45.5)	110 (39.5)	1	1
>5 years	76 (54.7)	168 (60.5)	1.27 (0.907, 2.103)	1.58 (1.482, 2.437)^∗∗^

Note: ^∗^*p* value > 0.01, ^∗∗^*p* value < 0.001, and 1 reference category.

## Data Availability

All the data were included in the study, and data will be available upon a responsible request to the corresponding author.

## Abbreviations

AOR: Adjusted odd ratio
CI: Confidence interval
COR: Crud odd ratio
EBP: Evidence-based practice
HO: Health officer
SPSS: Statistical Package for the Social Sciences
VIF: Variance inflation factor
WHO: World Health Organization.
